# Transatrial repair of a giant left ventricular pseudoaneurysm and ischaemic mitral regurgitation after myocardial infarction: case report

**DOI:** 10.1093/ehjcr/ytae397

**Published:** 2024-08-02

**Authors:** Yuki Monden, Dai Une, Megumi Furuta, Kenji Yoshida, Mikizo Nakai

**Affiliations:** Department of Cardiovascular Surgery, Okayama Medical Center, 1711-1 Tamasu, Kitaku, Okayamashi, Okayama 701-1192, Japan; Department of Cardiovascular Surgery, Okayama Medical Center, 1711-1 Tamasu, Kitaku, Okayamashi, Okayama 701-1192, Japan; Department of Cardiovascular Surgery, Okayama Medical Center, 1711-1 Tamasu, Kitaku, Okayamashi, Okayama 701-1192, Japan; Department of Cardiovascular Surgery, Okayama Medical Center, 1711-1 Tamasu, Kitaku, Okayamashi, Okayama 701-1192, Japan; Department of Cardiovascular Surgery, Okayama Medical Center, 1711-1 Tamasu, Kitaku, Okayamashi, Okayama 701-1192, Japan

**Keywords:** Cardiac surgery, Left ventricular pseudoaneurysm, Acute coronary infarction, Mitral valve replacement, Case report

## Abstract

**Background:**

Left ventricular pseudoaneurysm (LVPA) is an infrequent but highly lethal complication of myocardial infarction. Early surgical repair with a resection of pseudoaneurysm is often performed, given that medical therapy alone is associated with a high risk of mortality. This report describes a case of a giant LVPA on the lateral wall post-infarction and mitral valve regurgitation that was successfully treated by surgical transatrial closure and mitral valve replacement.

**Case summary:**

A 77-year-old man with chronic kidney disease and a history of percutaneous coronary interventions for acute myocardial infarction was referred to the cardiac surgeons because of a spontaneous finding of an abnormal mass adjacent to the heart on imaging studies, which was missed on a chest radiograph obtained 3 months earlier. Cardiac studies revealed LVPA and severe mitral regurgitation with poor ejection fraction. Early repair of LVPA and concurrent mitral valve surgery were recommended. Transatrial patch closure and mitral valve replacement were performed using an interatrial approach via median sternotomy. Although the patient’s post-operative course was complicated by congestive heart failure and irreversible renal failure, he was discharged with good functional status after 1 month of intermittent renal replacement therapy with haemodialysis.

**Discussion:**

Transatrial repair of LVPA and concurrent mitral valve replacement can be a treatment of choice for reducing surgical trauma to the left ventricle and protecting the sealing structure from rupture.

**Learning points**

- Left ventricular pseudoaneurysm (LVPA) is an extremely rare but highly lethal complication of myocardial infarction.
- Patients with acute myocardial infarction who have multiple risk factors for LVPA should be followed up for LVPA by auscultation or chest radiographs at a minimum.
- Transatrial repair of LVPA on the lateral wall with mitral valve replacement reduces surgical trauma to the left ventricle and protects the sealing structure from rupture.

## Introduction

Left ventricular pseudoaneurysm (LVPA) is an infrequent but highly lethal complication of myocardial infarction.^1^ While a conservative approach should be considered for a small aneurysm, surgical repair is the treatment of choice.^2,3^ Ventriculotomy and patch closure via the free wall have often been performed for LVPA repair, having been reported with high mortality rates.^2^ High-grade mitral regurgitation is common in patients presenting with left ventricular aneurysm, which is recommended to be corrected concurrently with aneurysm repair.^4^ This report describes a case of a patient with LVPA on the lateral wall with severe mitral regurgitation after percutaneous coronary intervention that was treated successfully using a transatrial approach.

## Summary figure

**Table:** 

Day 0	A 77-year-old man presented with chest discomfort that was diagnosed as acute coronary infarction, and he underwent percutaneous coronary intervention to the left circumflex artery. Post-interventional cardiac studies showed good cardiac function without any valvular disease or left ventricular pseudoaneurysm (LVPA)
Day 22	The patient was discharged home
Day 100	He re-presented with chest discomfort, which was diagnosed as unstable angina. He underwent percutaneous coronary intervention for residual stenosis in the left anterior descending artery
Day 111	He was discharged home despite evidence of an abnormal mass on a chest radiograph
Day 206	He presented to the outpatient clinic for an annual check-up of chronic kidney disease with kidney stones and was referred to the cardiac surgeons because of an incidental finding of an abnormal mass adjacent to the heart on imaging studies. Cardiac studies revealed LVPA and severe mitral regurgitation with poor ejection fraction
Day 216	Transatrial patch repair of LVPA and mitral valve replacement were performed. Perioperative management was well tolerated
Day 225	Reduced cardiac function was noted, and he was referred to the cardiologists for optimal medical therapy
Day 239	He developed congestive heart failure, which resulted in irreversible kidney dysfunction
Day 275	After 1 month of intermittent haemodialysis, he was discharged home with a good functional status

## Case presentation

**Figure 1:**
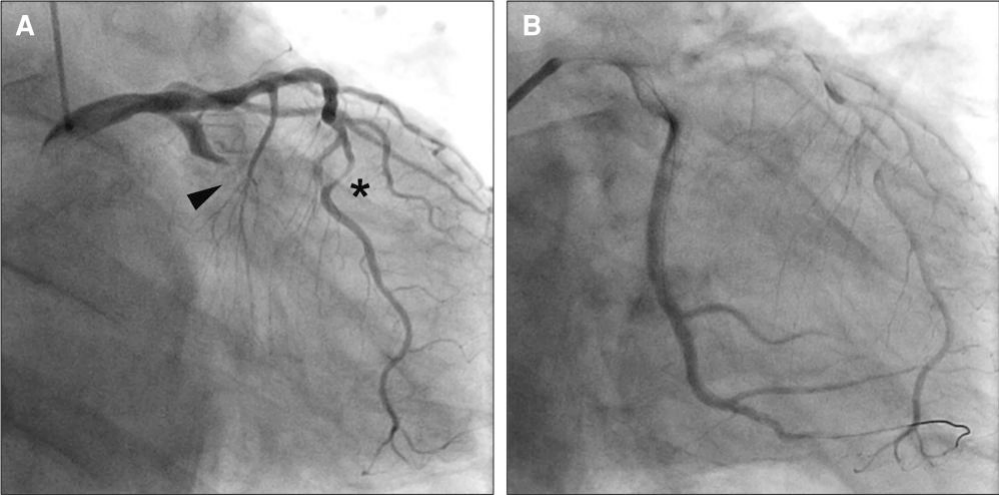
Emergent coronary angiography performed at the first presentation. (*A*) A proximal obstruction of the circumflex artery (black arrow) and significant stenosis in the left anterior descending artery (asterisk), but no significant lesions in the right coronary artery. (*B*) Reperfusion was achieved soon after subsequent percutaneous coronary intervention to the circumflex artery but with a significant delay in the flow of contrast.

A 77-year-old man with chronic kidney disease, renal stones, and Type 2 diabetes presented in the emergency department complaining of chest discomfort. Emergent coronary angiography revealed a proximal obstruction in the circumflex artery and significant stenosis in the left anterior descending artery but no significant lesions in the right coronary artery (*Figure 1A* and see Supplementary material online, *Video S1*). The circumflex artery was treated by percutaneous coronary intervention with an implantation of a drug-eluting stent, which achieved reperfusion but with a significant delay in flow of contrast (*Figure 1B*). Because of this reperfusion status, the cardiologists decided to monitor the patient carefully in the hospital setting for a longer time than usual. A post-intervention transthoracic echocardiogram showed preserved cardiac function without any significant valve disorders or aneurysm formation (see Supplementary material online, *Video S2*). The patient was discharged home 22 days after the intervention.

**Figure 2:**
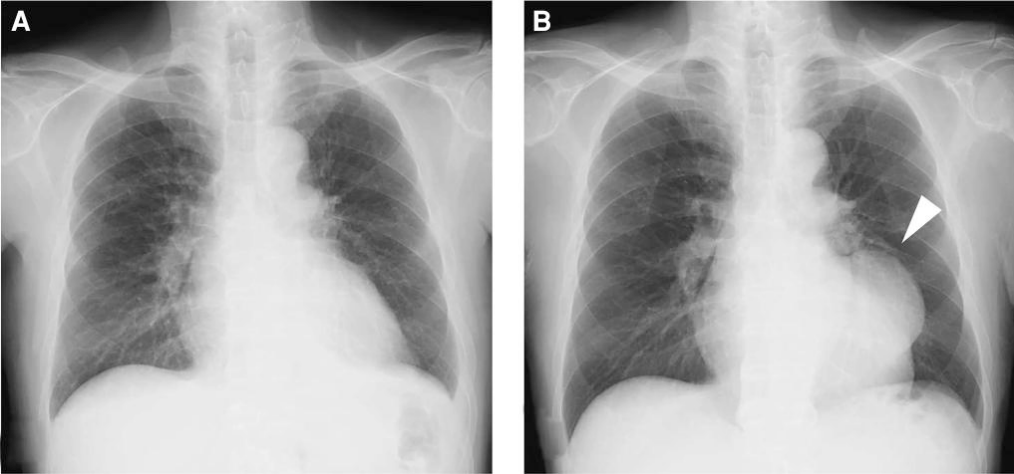
Chest radiographs. (*A*) At the first presentation. (*B*) At discharge after the second intervention showing a mass suspicious for left ventricular pseudoaneurysm (arrow).

Two months later, he presented to the outpatient clinic complaining of exertional chest discomfort. He was diagnosed with stable angina pectoris and underwent drug-eluting stent implantation for stenosis in the left anterior descending artery. The circumflex treated 2 months previously remained patent and did not require further intervention. After the intervention, he complained of reduced appetite, which required time to address before discharge. Two weeks later, he was discharged home despite the presence of an abnormal mass on a chest radiograph (*Figure 2*).

**Figure 3:**
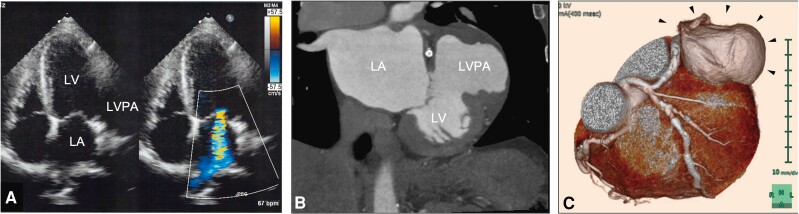
Cardiac imaging studies. (*A*) A transthoracic echocardiogram showing a huge aneurysm involving the lateral wall of the left ventricle with high-grade mitral valve regurgitation. (*B*) Cardiac computed tomography angiography revealing a contrast-filled outpouching on the lateral wall of the left ventricle. (*C*) A three-dimensional multiplanar reconstruction of the left ventricular pseudoaneurysm (black arrows). LA, left atrium; LV, left ventricle; LVPA, left ventricular pseudoaneurysm.

Seven months after the first presentation, he presented to the outpatient clinic for an annual check-up of his chronic kidney disease and renal stones. On auscultation, a new 3/6 systolic murmur was present at the apex of the heart. Computed tomography without contrast revealed a mass measuring 5 cm × 6 cm × 8 cm adjacent to the heart, which prompted a referral to the cardiac surgeons. A transthoracic echocardiogram showed a huge aneurysm involving the lateral wall of the left ventricle, severe ischaemic mitral regurgitation, and an ejection fraction of 35% (*Figure 3A*). Cardiac computed tomography revealed patent stents in both the circumflex and the left anterior descending arteries but also showed a contrast-filled outpouching on the lateral wall of the left ventricle (*Figure 3B* and *C*). Cardiac magnetic resonance imaging showed an aneurysm involving the adjacent cardiac and non-cardiac structures, which raised suspicion for LVPA rather than a true aneurysm. The orifice of the LVPA was located immediately adjacent to the mitral valve structure and measured 3.8 mm × 2.9 mm, indicating that transcatheter closure would be technically challenging.^3,5^ Given the nature and size of the LVPA, surgical repair was recommended in view of the high risk of rupture and mortality.^1^

**Figure 4:**
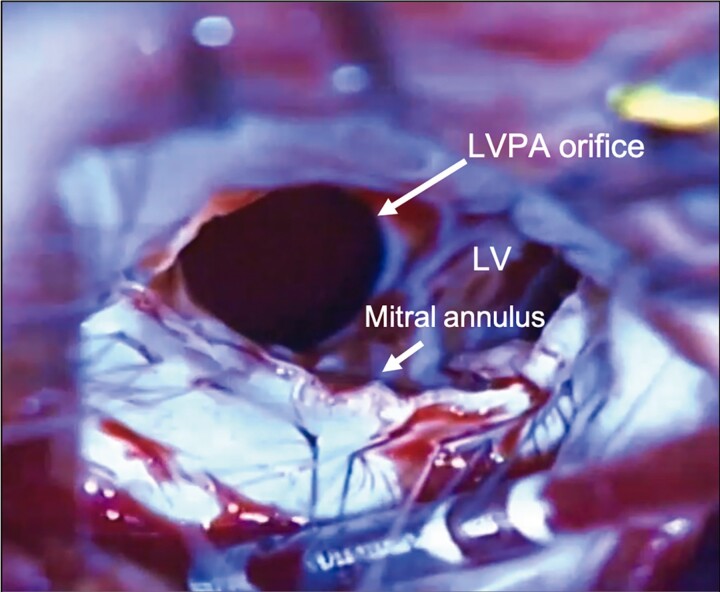
A surgeon’s view for repair of the left ventricular pseudoaneurysm. A transatrial view of patch closure with posterior leaflet resection showing an aneurysm with an orifice measuring 3.5 cm × 2.5 cm located between the mitral annulus and the base of the medial papillary muscle. LV, left ventricle; LVPA, left ventricular pseudoaneurysm.

Cardiopulmonary bypass was established via median sternotomy. A mass on the lateral wall was extensively adherent to the surrounding pericardium. After the induction of cardioplegia, a left atriotomy was made behind the interatrial groove, which revealed severe mitral valve regurgitation with a maladaptation of the leaflets and ruptured mitral chordae. The orifice of the aneurysm measured 3.5 cm × 2.5 cm and was located between the mitral annulus and the base of the medial papillary muscle behind the posterior leaflet (*Figure 4*). Considering the unsatisfactory view for patch closure, the complexity of the repair, and the ischaemic aetiology, we resected the posterior leaflet of the mitral valve and opted for mitral valve replacement instead of repair. After transatrial patch closure of the orifice, in which the posterior papillary muscle was included in the suture line, we proceeded to artificial chordal reconstruction and mitral valve replacement with an implantation of a bioprosthesis. Two artificial chordae were sutured on the anterior papillary muscle; one was placed on the anterior commissure and the other in the P2 position on the sewing cuff of the implanted bioprosthesis.

The patient was then weaned successfully from cardiopulmonary bypass. Acute post-operative management was well tolerated. Post-operative echocardiography confirmed no residual leak into the aneurysmal sac and a well-functioning bioprosthetic valve (see Supplementary material online, *Video S3*). He was referred to the cardiologists on post-operative Day 9 to optimize medical therapy for reduced cardiac function. Two weeks later, he developed congestive heart failure and irreversible renal failure requiring renal replacement therapy. After 1 month of inpatient renal replacement therapy, he was discharged on intermittent haemodialysis with a good functional status that was maintained at his 6-month follow-up visit.

## Discussion

Left ventricular pseudoaneurysm is a serious complication of acute myocardial infarction (AMI) and has a very poor prognosis.^1^ Left ventricular pseudoaneurysm has variable clinical signs and may be asymptomatic, making early diagnosis challenging.^1^ Myocardial infarction is the most common aetiology of LVPA, and patients who are older, male, and hypertensive and those who have inferior or lateral AMI are at a higher risk of developing LVPA post-infarction.^2,6^ However, the incidence of LVPA after ST-elevation myocardial infarction is extremely rare, with an estimated incidence rate of <0.3%.^2^ While true aneurysm includes both the endocardium and the myocardium, the wall of a pseudoaneurysm contains only fibrous tissue or pericardium. An LVPA is formed when a free wall rupture is contained by the surrounding pericardium and scar tissue and does not involve the true layers of the ventricle, namely, the endocardium and myocardium.^7,8^ The orifice of a pseudoaneurysm is more likely to be on the lateral or posterior wall than on the anterior wall, possibly because the anterior wall is more mobile, making an anterior wall rupture less likely to be sealed by the surrounding tissues and more likely to be fatal.^2,3^ While an untreated pseudoaneurysm had an ∼40% risk of fatal rupture, the mortality rate has been reported to be ∼20% in patients who undergo surgery.^2^ Therefore, surgical repair is the treatment of choice for LVPA, and a conservative approach should be considered only in specific patients, such as those with a small aneurysm.^2,3^

As with a true aneurysm, LVPA is often repaired by a resection of the pseudoaneurysm with patch closure through the free wall.^2,9–12^ It has been recommended that high-grade mitral regurgitation, which is common in patients presenting with left ventricular aneurysm, be corrected concurrently with aneurysm repair.^4^ The recently described transatrial approach is expected to reduce surgical trauma to the left ventricle.^10,13^ Given that LVPA is vulnerable to re-rupture and is already sealed by surrounding structures,^7,10^ we believe that transatrial repair, which preserves the sealing structures, is preferable to a resection of the pseudoaneurysm, which requires a manipulation of pericardial adhesions and removal of the sealing structures.

Although LVPA closure with off-label use of a percutaneous device has recently been reported, device closure for a large orifice (i.e. >20 mm) may be less feasible and is associated with a high risk of residual leak into the aneurysmal sac and serious complications.^3,5^ Therefore, surgery should still be considered as the standard management for LVPA, especially for an LVPA with a large orifice.^3,5^

A transatrial approach for LVPA repair often requires a partial resection of the mitral leaflets to gain a satisfactory view for closure because the LVPA orifice is often situated just below the mitral leaflet or annulus.^10,12,13^ Although a partial resection of the mitral leaflets can provide a satisfactory view and allow direct closure, repair after LVPA closure can occasionally require mitral valve replacement, even in patients without pre-operative mitral regurgitation.^10,13^ In terms of functional ischaemic mitral regurgitation, it has been demonstrated that there is no significant difference in left ventricular reverse remodelling or survival at 12 months between patients who undergo mitral valve repair and those who undergo replacement.^14,15^ Furthermore, the papillary muscle and chordae need to be involved in the patch closure line, indicating that complex mitral repair has doubtful durability. Therefore, although mitral valve repair with a resection of the leaflets can be feasible for pre-operative mitral regurgitation, we recommend mitral valve replacement rather than repair after LVPA closure.

## Conclusion

In this study, we have encountered a patient with LVPA that had been overlooked on an earlier chest radiograph and severe mitral regurgitation, who was treated successfully by surgical repair and mitral valve replacement. This report highlights the need for careful radiographic follow-up for LVPA post-infarction and the fact that a transatrial approach for LVPA closure and subsequent mitral valve replacement should be the treatment of choice. This surgical strategy can reduce surgical trauma, prevent rupture of the sealing structure, and provide a long-term benefit in terms of mitral function in patients who undergo mitral valve repair.

## Lead author biography


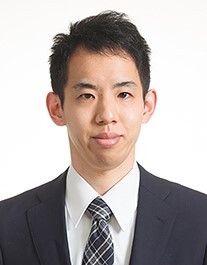


Dr Yuki Monden attended Tokyo Medical and Dental University for Medical School, did his internship at Shizuoka General Hospital, and is doing a general surgery residency at the Okayama Medical Center.

## Supplementary Material

ytae397_Supplementary_Data

## Data Availability

All data relevant to this case will be shared by the corresponding author upon reasonable request.
